# Biomimetic synthetic test system based on hydroxyapatite cement for adhesive strength evaluation of experimental mineral-organic bone adhesive materials

**DOI:** 10.1177/08853282241283537

**Published:** 2024-09-09

**Authors:** Paul Frederik Otto, Sebastian Hienz, Silvia Mittmann, Niklas Dümmler, Tobias Renner, Csaba Gergely, Juliane Carolin Kade, Uwe Gbureck

**Affiliations:** Department for Functional Materials in Medicine and Dentistry, 9190 University Hospital Würzburg, Würzburg, Germany

**Keywords:** Bone adhesive material, calcium phosphate cement, magnesium phosphate cement, phytic acid, NCO-sP(EO-stat-PO)

## Abstract

The development of bone adhesive materials is a research field of high relevance for the advancement of clinical procedures. Despite this, there are currently no material candidates meeting the full range of requirements placed on such a material, such as biocompatibility, sufficient mechanical properties and bond strength under biological conditions, practical applicability in a clinical setting, and no adverse effect on the healing process itself. A serious obstacle to the advancement of the field is a lack in standardized methodology leading to comparable results between experiments and different research groups. Natural bone samples are the current gold-standard material used to perform adhesive strength experiments, however they come with a number of drawbacks, including high sample variability due to unavoidable natural causes and the impossibility to reliably recreate test conditions to repeat experiments. This paper introduces a valuable auxiliary test method capable of producing large numbers of synthetic test specimens which are chemically similar to bone and can be produced in different laboratories so to repeat experiments under constant conditions across laboratories. The substrate is based on a hydroxyapatite forming cement with addition of gelatine as organic component. Crosslinking of the organic component is performed to improve mechanical properties. In order to demonstrate the performance of the developed method, various experimental and commercial bone/tissue adhesive materials were tested and compared with results obtained by established methods to highlight the potential of the test system.

## Introduction

Bone adhesive materials hold tremendous potential for revolutionizing orthopaedic procedures by improving surgical techniques, simplifying complex operations, and enhancing patient outcomes.^1–5^ They are specifically designed substances that bond with bone tissue, providing stability and strength during orthopaedic procedures. These materials are generally composed of biocompatible compounds that should ensure minimal adverse reactions and promote successful bone healing, although most materials currently available or in clinical use cannot meet every aspect of the complex challenges that need to be addressed simultaneously for optimum results.^1,6,7^ Some common types of experimental bone adhesives include cyanoacrylate- and methacrylate-based systems as well as protein or polysaccharide materials.^8–20^

In recent years, magnesium phosphate cement (MPC) has emerged as a promising material for bone adhesive applications due to its excellent biocompatibility, bioactivity, and bonding capabilities.^21,22^ In contrast to the usual lack of adhesive properties in mineral cement-based materials, by changing the setting mechanism of MPC to involve a chelating reaction with an agent such as phytic acid (IP6), strong adhesive bonds to bone surfaces can be achieved.^21,23,24^ A similar material platform based on tetracalcium phosphate (TTCP) and phosphoserine (OPLS) has lately received much attention as one of the first mineral cement based adhesive material approaching market readiness.^25–31^

The testing of adhesive properties of novel materials intended for use in the human body is challenging, because simulating the environment these materials are subjected to through their intended use is hard to get right without introducing additional problems. Researchers face difficult choices in experimental design regarding their choice of substrate material and testing conditions. Understanding these challenges is crucial for the development and optimization of MPC-based bone adhesives, ultimately leading to improved clinical outcomes and patient care. In scientific practice, a large number of non-uniform and often hard-to-repeat procedures are used to provide quantitative results for newly developed adhesive materials, a problem which inspired a number of studies trying to find improved and more reliable methods.^32–35^

One very common approach is the use of bone from natural sources. The obvious and valid reasoning behind this is that only by testing on the intended substrate, reliable predictions of adhesive performance can be made. In smaller studies, this benefit might make them an attractive choice for experimental design. Nevertheless, this approach has several drawbacks: Individual samples are often cut from different sections or areas of the bone, and the bones themselves are sourced from different animal individuals with unknown age and health. Further variations are caused by delivery times, freezing cycles and the drift introduced in the samples by lengthy preparation times at elevated temperatures, especially if larger studies are conducted.^36^

These challenges and the fact that only limited numbers of sufficiently similar sample specimens can be prepared in a reasonable amount of time severely limit the research opportunities for bone adhesive development. Even the repetition of a given experiment or conducting a larger number of tests over the course of months and years cannot compensate that, as results produced in such a manner are usually very hard to compare since constant sample conditions can never be assumed with sufficient confidence. While some of these problems can be addressed by researchers performing a study, issues with comparability remain for colleagues in the field reading and building on their work.

In this paper, a bone adhesive test system is introduced to overcome the previously mentioned drawbacks and consisting of synthetic materials while mimicking the chemical composition of bone with its inorganic and organic components to produce meaningful results. The production of large amounts of samples is enabled, and experiments can be repeated under the same conditions in different laboratories and at various time points by relying on simple compounds which are commercially available worldwide.

The sample specimens are formed by an α-tricalcium phosphate (α-TCP) cement setting to form calcium deficient hydroxyapatite (CDHA), which is close to identical to the main mineral component of natural bone, where hydroxyapatite (HAp) with some degree of substitution by biologically relevant ions is prevalent. The composition of natural bone can be stated as roughly 65% mineral (HAp), 25% organic (mostly proteins, about 90% of these being collagen I) and 10% water.^37^ The organic component was introduced in the test system specimens by adding varying amounts of gelatine to the cement formulations, with the aim to find paste compositions able to form stable test specimens with a composition close to natural bone. To improve the mechanical stability of test specimens containing gelatine and solve issues associated with testing very strong adhesives on weaker substrates, experiments on crosslinking the organic phase and its effect on adhesive strength tests have been performed as well. In the context of cement preparation, the gelatine content was defined as the mass of gelatine contained in the final test specimen relative to its dry mass and can be calculated directly from the cement paste composition.

## Materials and methods

### Powder synthesis

TTCP (Ca_4_(PO_4_)_2_O) and farringtonite (Mg_3_(PO_4_)_2_) powders used as part of adhesive compositions were prepared by sintering, whereas highly reactive (Nr. 2933) magnesium oxide (MgO) was purchased from Magnesia GmbH (Lüneburg, Germany), and α-TCP (Ca_3_(PO_4_)_2_) for test specimen preparation from Innotere (Radebeul, Germany).

TTCP powder was synthesized from a 1:1 molar ratio of dicalcium phosphate (CaHPO_4_) (Fluka Honeywell, New Jersey, United States) and calcium carbonate (CaCO_3_) (Merck, Darmstadt, Germany) by sintering at 1500°C for 5 h. Farringtonite powder was prepared from dimagnesium phosphate (MgHPO_4_) (Sigma-Aldrich/Honeywell, Missouri, United States) and magnesium hydroxide (MgOH) (VWR Prolabo, Pennsylvania, United States) in a 2:1 molar ratio. The magnesium hydrogen phosphate was sieved to ≤125 µm and ground in an agate jar for 1 h at 200 r/min, mixed with magnesium hydroxide and sintered at 1100°C for 5 h. Prior to adhesive preparation, the powders were sieved to <125 μm (TTCP) and ≤355 µm (farringtonite), respectively.

### Test specimen preparation

**Figure 1.**
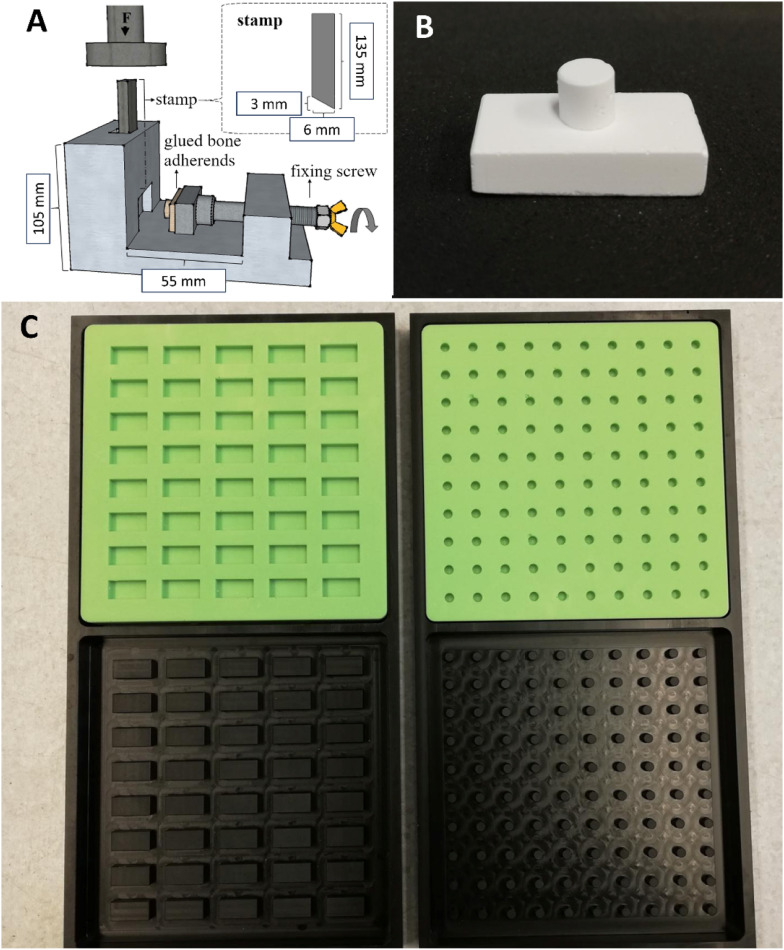
(A) Test setup for adhesive strength experiments as previously presented by Renner for natural bone samples.^39^ (B) Test specimens fit for the same setup prepared from cement pastes using (C) silicone forms (green) cast with precisely machined negative forms (black).

Mineral and mineral-organic cement pastes for the preparation of adhesive and compressive strength test specimens were prepared by mixing α-TCP powder sieved to <120 µm with 5% Na_2_HPO_4_-solution in varying powder-to-liquid ratios (PLR [g/mL]: 2, 3, 4, and 5). Cold water fish skin derived gelatine (Sigma-Aldrich, the Netherlands) was dissolved in the Na_2_HPO_4_-solution prior to the addition of α-TCP powder to promote homogeneous distribution. Gelatine concentrations of 10 wt%, 20 wt%, 30 wt%, and 40 wt% were evaluated. A full overview of all prepared combinations is provided in Table S1 in the supporting information. The pastes were moulded into cuboidal test specimens of 20 × 10 × 5 mm and cylindrical test specimens (d = 5 mm, h = 5 mm) for adhesive strength tests or cuboidal test specimens of 12 × 6 × 6 mm for compressive strength tests, respectively (see Figure 1(B)). The moulds themselves were cast with fast-curing silicone (Dublisil®) in negatives that are CNC-milled from durable plastic as seen in Figure 1(C). The moulds were left for initial hardening at 37°C and 100% relative humidity for 1 h. The specimens were then demoulded and transferred into aqueous solutions for a longer setting period as discussed in the results section.

To improve the mechanical properties of the mineral-organic test specimens, the organic phase was replaced by a cross-linkable derivate and an initiator system. It consisted of gelatine methacryloyl, which was synthesized from cold water fish skin derived gelatine (Sigma-Aldrich, the Netherlands) according to established procedures.^38^ 5 wt% ammonium persulfate ((NH4)2S2O8) was dissolved in water first and 0.1 wt% tetramethyl ethylene diamine (C6H16N2) was added to the final cement liquid to induce crosslinking of fish derived gelatine methacryloyl (GelMA-F) during the setting reaction. These specimens were prepared at PLR 4 g/mL with a GelMA-F concentration of 30 wt% in solution.”

Bone test specimens were prepared from the diaphysis of fresh bovine femurs. Muscle and tendon attachments, periosteum, and bone marrow were removed. Pieces of cortical bone were cut with a handsaw and shaped with SiC wet-grinding paper (grit 80) under water-cooling to obtain cuboidal test specimens of about 20 × 10 × 5 mm. Cylindrical test specimens (d = 5 mm, h = 5 mm) were shaped with a Robling 800 lathe at 2000 r/min. The test specimens were stored in phosphate buffered saline (PBS) at 5°C until use.

### Adhesive strength tests

Test specimens were taken from their setting solutions or, in case of bone, PBS and excess fluid was removed with a laboratory cloth. The samples were then immediately glued together in a moist state, as indicated in Figure 1(B). The various adhesives were processed according to their respective requirements, but always within the same time frame of 1–3 min at a controlled laboratory atmosphere of 21 ± 1°C. The preparation and mixing varied for different adhesives, where commercial protein-based materials (BioGlue®, CryoLife, USA) and (Tisseel® fibrin glue, Baxter, Germany) were applied via dedicated mixing cartridges, cyanoacrylate (TruGlue®, Trusetal, Germany) was applied immediately, and cement-based adhesives were quickly prepared with a spatula on a glass plate as intended and applied with a syringe.^21,26,39^ The adhesives were let to form an initial bond for 10 min at 100% relative humidity and 37°C. To study the degradation of the samples, bonded specimens were immersed in PBS for 2 h or 24 h at 37°C prior to mechanical tests.

Shear strength testing was performed using a mechanical testing machine (Zwick/Roell Z010, Zwick GmbH & Co. KG, Ulm, Germany) at a crosshead speed of 1 mm/sec and a pre-load of 1 N in a setup as shown in Figure 1. The bonded sample was sheared with a force applied parallel to the bonded surface until failure. The adhesive strength was calculated from the maximum force measured at the time of failure and the bond surface area of approx. 20 mm^2^.

### X-ray diffraction analysis (XRD)

The phase composition of the cement powders and test specimens was analysed using a D8 Advance X-ray diffractometer (Bruker Company, Billerica, United States) with CuKα radiation at an accelerating voltage of 40 kV in an angular range of 10°–70°. Measurements were made at a step rate of 1.2 s/step and a step size of 0.02°. Phase identification and Rietveld refinement to determine the quantitative phase composition were performed with DIFFRAC.EVA and DIFFRAC.TOPAS softwares (Bruker, Billerica, United States) using reference patterns from the International Centre of Diffraction Data database (PDF-2, 1996) for farringtonite (PDF 33-0876), magnesium oxide (PDF 43-1022 and PDF 04-0829), tetracalcium phosphate (PDF 25-1137), and α-tricalcium phosphate (PDF 29-0359).

### Hg porosimetry

The open porosity of the test specimens was determined by mercury porosimetry (PASCAL 140/440, Porotec GmbH, Hofheim, Germany). Prior to measuring, the test specimens were stored and dried for 24 h at 37°C in a drying oven to remove any excess water from the pores.

### Fourier transform infrared spectroscopy (FTIR)

Infrared spectra of the raw materials and finished test specimens were obtained by Fourier Transform Infrared Spectroscopy (FT-IR; Nicolet is10, Thermo Scientific, Waltham, MA) in a range from 400 cm^−1^ to 4000 cm^−1^ with a spectral resolution of 4 cm^−1^.

### Statistical analysis

The software SigmaPlot (Systat Software GmbH, Erkrath) was used for statistical analysis of the experimental data sets. The Shapiro-Wilk test was used to check the sets for normal distribution. One-way ANOVA or Kruskal-Wallis One-Way Analysis of Variance on Ranks were used to evaluate significance, depending on the data set. Pairwise comparison was performed using the Holm-Sidak Method or Dunn’s Method. The significance level was defined as follows: *p* > .5, *p < .5; **p < .01; ***p < .001, where indicated in the figures.

## Results and discussion

### Powder characterization

Several cement powder compositions were synthesized and used throughout this work for the preparations of mineral and mineral-organic adhesive materials and the synthesis of biomimetic test specimens. Basic characterization of these powders for the purpose of ensuring consistent quality includes X-ray diffraction analysis to determine phase composition and particle size distribution analysis after ball milling. The results are shown in Table 1. Corresponding X-ray diffractograms of all cement powders used in this work are provided in the supporting information (Figure S1 & 2).

**Table 1.** Powder characterization.

Phase composition (XRD)		Particle size (µm)		
		d_10_	d_50_	d_90_
α-TCP	>99% α-TCP	59.9	16.1	1.8
Farringtonite	>99% Farringtonite	16.7	7.6	1.8
MgO	99% Periclase; 1% Brucite	23.5	11.4	2.1
TTCP	94% Hilgenstockite; 6% α-TCP	141.6	39.6	25.7

### Test specimen compositions

Two approaches were used in parallel in this work: A simplistic, purely mineral test specimen type was produced by letting a standard α-TCP cement set to form calcium deficient hydroxyapatite. To match the chemical composition of natural bone, gelatine solution was incorporated in the cement paste formulation to form mineral-organic specimens in a more complex approach, providing more realistic insights into the mechanism of bone adhesives. Gelatine solution concentrations and PLRs were varied to obtain reasonable gelatine contents in the finished test specimens while maintaining acceptable paste consistency as well as final mechanical properties. Here, we used gelatine from cold-water fish skin, which is capable of forming sufficiently thin pastes even at room temperature in elevated concentrations, whereas most commercially available products are made from bovine or porcine skin usually resulting in cement pastes that are difficult or impossible to process at high gelatine contents. This enabled the fabrication of mineral-organic specimens with gelatine contents as high as reported here, previously unachieved in usable α-TCP cement pastes.

**Figure 2.**
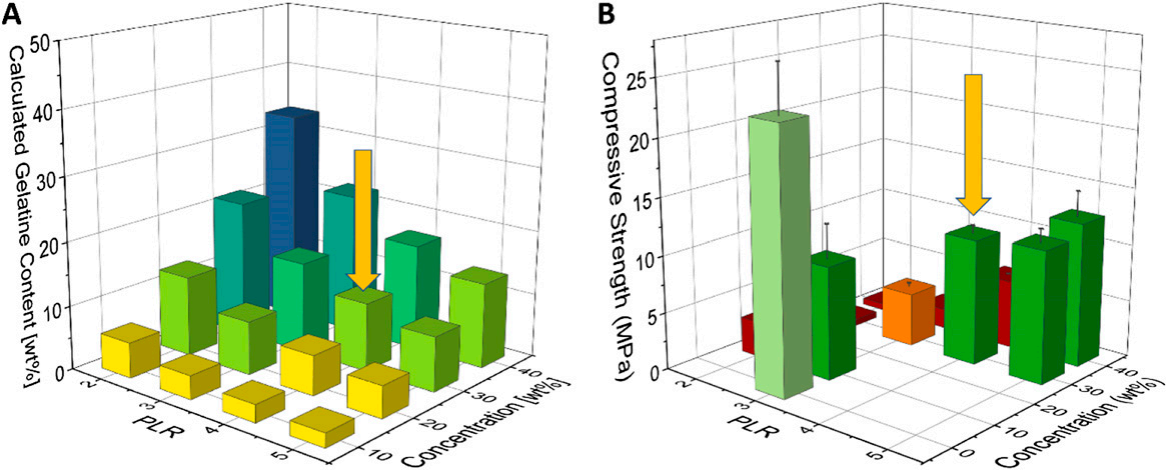
(A) shows the calculated gelatine content of set test specimens corresponding to the composition of the cement pastes used to prepare them and (B) the compressive strength shown by the resulting cements. Some low-gelatine compositions were not prepared. Concentration = 0 wt% corresponds to the purely mineral composition. The yellow arrow indicates the mineral-organic composition used for all further experiments. The exact values of the results presented here can be found in Table S1 in the supporting information.

Paste compositions with PLR varying from 2 to 5 g/mL and gelatine solution concentrations up to 50 wt% were prepared. The resulting compressive strength of the hardened cements was tested (Figure 2), with pure hydroxyapatite being the strongest at 22.8 MPa.

While specimens with a gelatine content of up to 33.3 wt% could be obtained, closely matching the composition of natural bone, these showed a stark deterioration of their mechanical properties in comparison. All compositions with gelatine contents above 15 wt% had weak compressive strength below 5 MPa (indicated by red shades in Figure 2(B)), unsuitable for adhesive strength tests as this indicates the specimens themselves might fail well before the adhesive does. Four compositions with similar compressive strength around 10 MPa (green in Figure 2(B)) and gelatine content ranging from 3.7% to 13.3% were found. The consistency of the cement pastes was used as secondary selection criterion for further experiments. Pastes at PLR 5 g/mL were observed to be very thick with a dry appearance. This caused them to be time-consuming to make and required special care while moulding to achieve accurate specimen geometries, making them less favourable for high-throughput studies. Thus, adhesive strength tests were performed on mineral-organic test specimens obtained from pastes with PLR 4 g/mL and a gelatine solution concentration of 30 wt%. This results in a gelatine content of 10.7% while maintaining an acceptable compressive strength of 11.0 MPa. While most adhesive materials could be tested well on this substrate, cyanoacrylate could cause issues due to its high final strength as will be presented and discussed in the adhesive strength test section. To address this, specimens with a crosslinked organic phase have been produced with the resulting improvements discussed in the last part of the paper.

**Figure 3.**
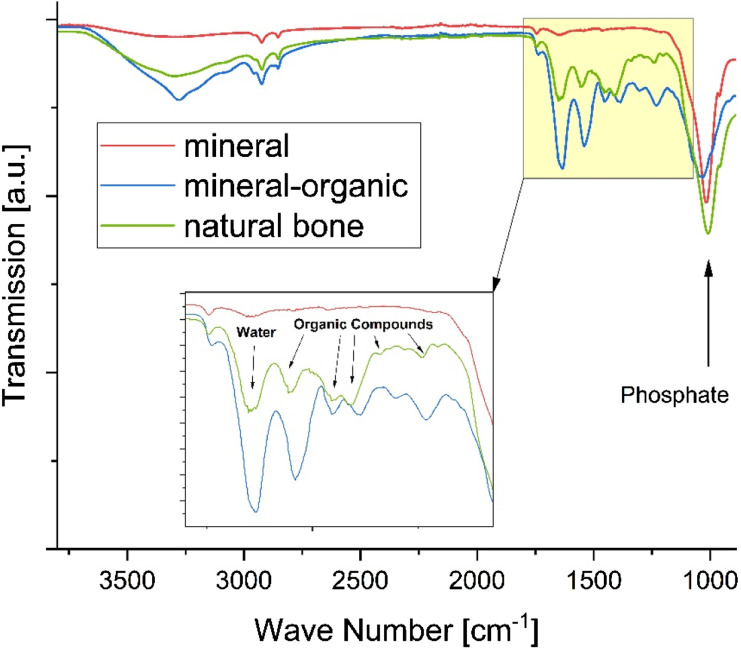
FTIR-spectra of cement-derived test specimens compared to natural bone. The detailed view in the inset shows a wave number interval containing the majority of characteristic signals of the protein components present in bone, which are closely approximated by the mineral-organic cement composition.

Figure 3 shows the FTIR spectra of set mineral and mineral-organic test specimen surfaces as well as a sample of bovine femur corticalis. The mineral test specimen has only one significant peak at 1012 cm^−1^ corresponding to PO_4_^2−^ of calcium phosphate, which is present in the mineral-organic specimen and in natural bone as well. In the area between 1100 cm^−1^ and 1800 cm^−1^, where many functional groups present in organic molecules have their characteristic peaks, a strong similarity of the FTIR spectra of natural bone and the mineral-organic test specimens can be observed. This supports the hypothesis that the presence of functional groups of organic compounds on the bone surface is adequately represented by the composition of the mineral-organic test specimens. Reference spectra for the α-TCP powder and gelatine solution used for preparation of the test specimens as well as a sample of commercially available hydroxyapatite powder are provided in the supporting information (Figure S3).

**Figure 4.**
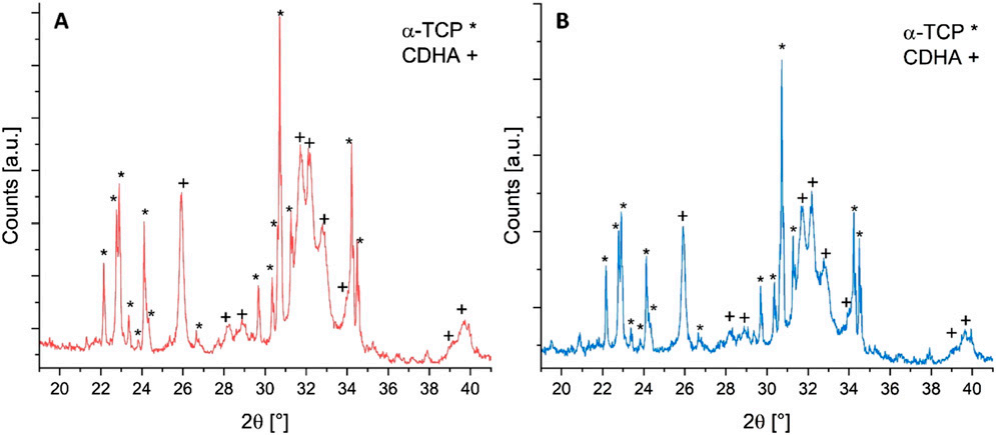
Diffractograms of (A) pure mineral and (B) mineral-organic specimens. While the conversion of α-TCP to hydroxyapatite is slightly decreased, no qualitative difference in the phase composition of the inorganic component is observed upon addition of gelatine to the cement.

The crystalline phase composition of the finished test specimens was determined via XRD analysis. Figure 4 shows the X-ray diffractograms corresponding to the set test specimens confirming the formation of hydroxyapatite.

A concern during the setting reaction of the cement (which traditionally takes place immersed in water or a salt solution at elevated temperatures over the course of several days) is to preserve the gelatine fraction, which might be dissolved at these setting conditions resulting in lower effective gelatine content on the surface in actual contact with the adhesive material, as well as an increase in porosity of the test specimens. Also, a decomposition of the gelatine must be avoided. Milder setting conditions were tested for mineral-organic specimens, with variation in setting time, setting temperature, and setting environment, as shown in Table 2. Rietveld refinement was used to quantify the conversion rate of the cement pastes to hydroxyapatite. Conversion rates of different test specimen groups are shown in Table 2 and pore size distribution plots obtained by Hg-porosimetry are supplied in the supporting information (Figure S4).

**Table 2.** Conversion rates of test specimens obtained under varying setting conditions.

Composition	Setting conditions	Conversion (%)
Mineral	7 days, 37°C, immersed	71
	7 days, 37°C, immersed	67
	3 days, 37°C, immersed	60
	3 days, RT, immersed	59
Mineral-organic	7 days, RT, immersed	64
	7 days, 37°C, humid air	54
	14 days, RT, immersed	70
	14 days, 37°C, humid air	53

The safest method to ensure no gelatine is leached from the test specimens is to change the setting environment from immersion in water to incubation in a humidity chamber at 37°C and 100% relative humidity to prevent the paste from drying out. Since the presence of water is vital as a transport medium for the reactants of the cement reaction, the conversion rate dropped to just over 50% using this approach, showing also that the water present in the paste is not sufficient to facilitate the reaction to the same extent as immersion. This detrimental effect could not be mitigated by extending the setting period up to 14 days. These samples were not selected for further experiments, as they had reduced structural integrity despite their lower porosity which is known to be a key factor for mechanical strength of brittle materials. The specimens had the lowest porosity of all obtained mineral-organic specimens, indicating that some leaching of gelatine content might have taken place in the other groups, although the differences are too small to be considered conclusive.

The mildest conditions with setting for 3 days at room temperature but still immersed in water resulted in specimens of adequate stability which were considered suitable for adhesive strength studies. All further experiments were conducted with specimens obtained under these conditions and compositions as detailed earlier in this section. Measures to prevent leaching of gelatine from the specimens during the setting reaction were investigated and are discussed at a later point in this work.

### Adhesive strength tests

Adhesive strength tests were performed on mineral and mineral-organic test specimens, as well as natural bone as reference, using a variety of adhesive materials as shown in Table 3. The performance of the two synthetic test systems was evaluated and compared to bone, but special attention was also paid to their individual advantages and disadvantages as well as possible synergy effects from the use of both systems.

**Table 3.** Names and sources of the adhesive materals used for adhesive strength evaluations in this study.

Name	System	Source
MPC-IP6	Magnesium phosphate cement	Prepared according to^21^
MPC-IP6-NCO	Magnesium phosphate cement with isocyanate additive	Experimental, derived from^21^
Tetranite®	Calcium phosphate cement with phosphoserine	Commercial track, patent^26^
BioGlue®	Albumin/Glutaraldehyde	Commercial (CryoLife, USA)
Tisseel®	Fibrin/Thrombin	Commercial (Baxter, Germany)
TruGlue®	Cyanoacrylate	Commercial (Trusetal, Germany)

**Figure 5.**
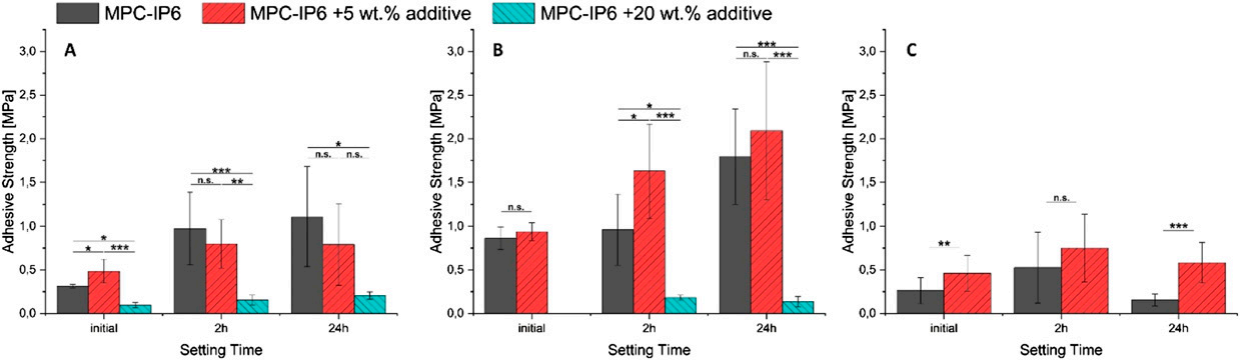
Adhesive strength test results of MPC-IP6 (with and without additive) on test specimens containing (A) only α-TCP versus (B) a gelatine content of ∼10 wt%. (C) Adhesive strength test results with two of these adhesive compositions on natural bone sourced specimens.

Figure 5 shows adhesive strength results for a previously described bone adhesive cement based on farringtonite and phytic acid (MPC-IP6) on the purely mineral as well as the mineral-organic test system. In an experimental approach, the adhesive cement is modified by a polymeric additive in two different concentrations (5 wt% and 20 wt%). The additive has several functional groups (isocyanates) designed to form covalent bonds to nucleophilic sites in biological compounds and thus improving its bond strength in a surgical environment as has previously been reported in a similar approach.^40^ Comparison of the results obtained on the different test systems reveals their predictive capabilities and limitations.

The addition of 20 wt% additive (blue) results in poor setting of the adhesive and was not repeated on natural bone. A clear improvement in adhesive strength is introduced by adding 5 wt% of a polymer-additive (red) to the MPC on a mineral-organic substrate whereas the adhesive strength on a purely mineral surface does not show this effect.

At high additive concentration of 20 wt%, the mechanical strength of the adhesive cement is compromised by the additive leading to universally low adhesive strength measurements uncorrelated to the bonded surface. At 5 wt% additive, the mineral test system indicates a decrease in adhesive strength, which could possibly be attributed to a less severe expression of the same effect, but no functionality is observed in the additive. In the case of the mineral-organic system, however, an increase in adhesive strength is observed, as here the chemical environment for the additive to develop its intended effect is comparable to the surface of bone for which it is ultimately designed. This increase in adhesive strength could be supported by a subsequent measurement performed on natural bone samples.

The use of the mineral-organic test system is more predictive for performance of materials on natural bone due to their chemical similarity and would thus be the recommended approach for any more specific study. Nevertheless, the mineral system is still able to provide intrinsically comparable results for adhesive materials not explicitly relying on mechanisms requiring an organic component to be present. In some cases, a careful comparison of results obtained from both systems might be able to provide insight into the mechanisms of other experimental adhesive compositions.

If degradation tests of the bonded specimens are carried out over a longer period of time (>7 days), specimens made of natural bone or mineral-organic test specimens show signs of degradation on the substrates themselves with a corresponding development of odour leading to doubts regarding the validity and ultimately to the rejection of the results. For a qualitative comparison of the long-term properties of different experimental adhesive materials, the use of the mineral test system could provide a way to still obtain some meaningful results by being able to ensure long-term tests under constant conditions, albeit with a chance to miss a specific effect and lesser general informative value compared to the other systems since the organic component is lacking. It is up to researchers to decide in their experimental design, what aspects of an experimental adhesive they need to focus on and therefore what they should prioritize when selecting a suitable time frame and substrate for testing. Negatives for casting the moulds can be freely designed and machined to fit the specifications of any custom or normed test-setup meeting the needs of various contexts.

**Figure 6.**
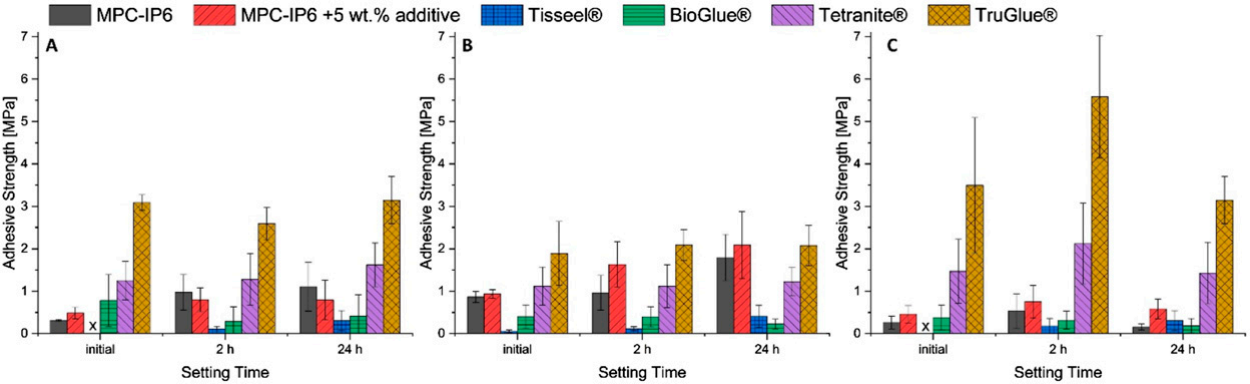
Adhesive strength measurement results for a variety of experimental and commercial bone adhesives on different test specimens (A) mineral (B) mineral-organic (C) natural bone samples.

Figure 6 shows an extensive study of several experimental adhesive compositions, as well as commercially available and clinically used tissue adhesives based on different material approaches. An overview is provided in Table 3. In addition to the previously mentioned magnesium phosphate cement with and without polymeric additive, a recently patented adhesive cement formulation termed Tetranite® based on tetracalcium phosphate is used.^26,27^ BioGlue® is a commercial tissue adhesive (CryoLife, USA) based on bovine albumin and glutaraldehyde, Tisseel® (Baxter, Germany) is based on fibrinogen and human thrombin. Fibrin adhesive is mainly used for gluing soft tissues during surgery to assist reducing blood loss. It has also been reported for application in osteochondral procedures.^41^ Its strength has been reported as roughly 2.5 kPa in a peel-away setup for skin flaps adhered to soft tissue.^42^ Though it is not originally intended as a bone adhesive material, it may be used as an ad hoc solution in surgery. Finally, the cyanoacrylate tissue adhesive TruGlue® (Trusetal, Germany) was tested as well.

Cyanoacrylate tissue adhesives are known for their high initial adhesive strength and possess a low viscosity allowing them to easily infiltrate porous material.^10^ In this study, this led to an increase in test specimens failing prior to the adhesive bond leading to a reduction in predicted adhesive strength in comparison to the actual capabilities of the adhesive. Generally, a limitation is placed on the maximum adhesive strength which can be resolved by the structural integrity of the test specimens themselves. From observations of such cases during the experimental procedures, the threshold above which reliable results can no longer be obtained lies at roughly 2–3 MPa. For adhesives intruding into the specimen’s porosity such as cyanoacrylates, this value might be lowered. This effect needs to be addressed, as it disproportionately lowers the predicted adhesive performance for a given material in comparison to one which does not exhibit this unintended mode of failure. In this study, this could result in the false assumption that CA is not much stronger than other tested materials. The true explanation lies in the fact that for this particular adhesive, the maximum resolvable adhesive strength was reached for both synthetic test systems. For any given study, it is important to assess whether this limitation will be relevant. Possible measures to improve these values are discussed in the following section.

### Crosslinking of organic component

Several concerns of the mineral-organic test systems could be addressed by chemically modifying the gelatine component of the cement pastes with methacryloyl groups, providing a functional site for crosslinking the gelatine. A chemical radical initiator system consisting of ammonium persulfate and tetramethyl ethylene diamine was used for crosslinking the organic component during the setting reaction of the cement.^38,43^

**Figure 7.**
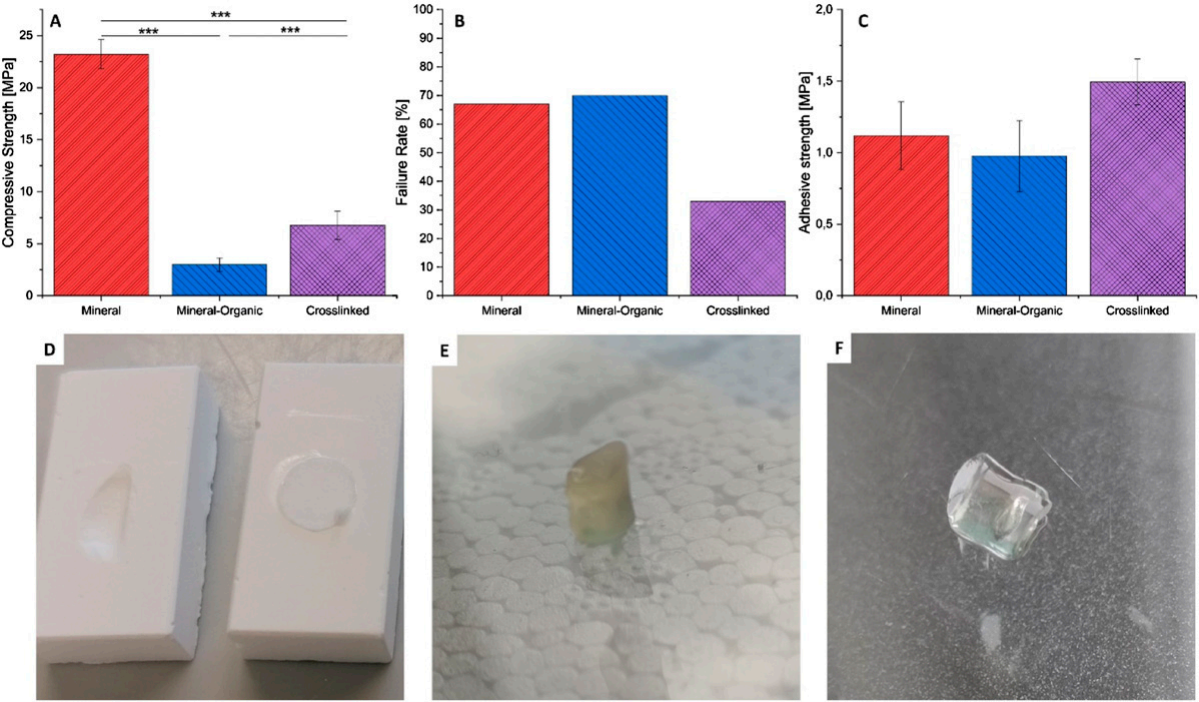
(A) Side-by-side comparison of compressive strength values for different cement compositions. Mineral-organic formulations are weaker, but a significant increase is achieved by crosslinking of the organic phase. (B) Setup intentionally designed for high failure probability (undegraded CA hardened for 24 h). Shown are the failure rate, which is the fraction of specimens failing before the adhesive joint does, and the mean calculated adhesive strength (C) when this occurred. The mineral-organic system performs slightly worse than the purely mineral in both categories, but crosslinking greatly improves the behavior in challenging conditions. (D) Mineral test specimens with adhesive failure of the joint on the right and specimen failure due to intrusion of CA on the left (unwanted under normal circumstances) (E) 30 wt% Fish derived GelMA samples crosslinked for 60 min in 100% rel. humidity at 37°C. This replicates the initial hardening conditions of test specimens without the inorganic component. (F) The same sample subjected to final setting conditions of the mineral-organic specimens (immersed in water at RT) showing no signs of dissolution.

Crosslinking effectively prevented dissolution of gelatine from the test specimens. While uncrosslinked test gels without cement dissolved, test gels of the crosslinked organic phase were found to be physically stable for 5 days (see Figure 7(E) and (F)) under the same conditions as the cement setting environment (RT in H_2_O), as well as the harsher adhesive degradation experiments (37°C in PBS). This ensures that the chemical composition of the specimen surface reflects the paste composition and constant properties of the specimens unaffected by the volume of the container used to harden the specimens due to saturation effects.

A successful modification with a crosslinked organic phase in the mineral-organic specimens could only be obtained by carefully matching the kinetics of the crosslinking reaction to the setting properties of the cement. While fast crosslinking is preferrable to ensure insolubility after 1 h, when the pre-hardened specimens are demoulded and transferred to their setting solution, attention must also be paid to the workability of the cement pastes. Too much initiator and catalyst resulted in fast increase of the viscosity of the cement pastes, not allowing proper sample production. 5 wt% ammonium persulfate and 0.1 wt% tetramethyl ethylene diamine were determined to be the optimum trade-off point, satisfying both requirements.

During shear strength experiments of adhesive materials, the test specimens themselves are subjected to forces that induce a complex combination of shear and tensile stress, which may lead to test specimen failure instead of the adhesive joint. If this occurs too frequently, the meaningfulness of the test is severely limited. In the previous section, this was shown to affect measurements obtained for cyanoacrylate adhesives. Basic compressive strength evaluation of the test specimen compositions can hint at the maximum resolvable adhesive strength but does not fully reflect the load distribution of an adhesive strength test. A more comprehensive measure for comparison of the test system performance is given by calculation of the mean adhesive strength measured in the cases of premature failure for any given specimen composition. The absolute value calculated this way does not indicate much as it can depend on the adhesive used, but it can be used to indicate whether a change in the test system composition improves its performance in this context. Additionally, it may be used as a rule of thumb: Adhesives capable of resulting in adhesive strength values well above this benchmark such as cyanoacrylate might be underestimated by the test, while not surpassing it much reliably yields usable results.

With this in mind, a shear strength test setup was designed to be likely to result in specimen failure by using cyanoacrylate hardened for 24 h without degradation. Figure 7 shows the mechanical properties and performance of different test system compositions under these conditions. While the compressive strength of the cement is substantially smaller for mineral-organic compositions, a significant increase can be achieved by crosslinking the organic phase during the setting reaction of the cement.

The increased toughness of the crosslinked cement helps reduce the failure rate (fraction of samples failing prematurely in each group) while using the same adhesive. Only a third of the samples fail after crosslinking, while for the mineral and uncrosslinked systems less than half of the specimens produce correct results. The results shows a tendency for higher calculated adhesive strength values in the event of a premature specimen failure for the crosslinked composition, while the overall likeliness of a failure is substantially decreased. Further improvements in the future could potentially obtained by a reduction of the test specimens’ porosity, as intrusion of low viscosity adhesives was observed to be a factor contributing to unintended failure modes. Multimodal particle size distributions are a promising way to address this approach.

## Conclusions

Adhesive strength tests of experimental bone adhesives can be improved by removing hard-to-track factors of variation induced by using natural bone as test substrate by replacing it with a synthetic material produced under controlled and simple conditions.

The work presented in this paper has demonstrated the production and applicability of two synthetic test substrates that are particularly tailored for the evaluation of bone adhesive materials. Two levels of complexity are explored, offering either a full-scale model with representation of bone surface chemistry, or simplicity in production and longevity by omitting the organic phase. Both presented test substrates offer high throughput and may have a place in scientific studies, with the complex mineral-organic system on one hand performing better in terms of predictive capabilities. With it, it was possible to predict the benefit of a polymeric additive in an experimental bone adhesive material, an effect that was also seen on natural bone samples but was not observed on the simpler, purely mineral system, which in turn provides easy access to higher sample numbers, long-term studies and possible insights in adhesive mechanisms through comparison of results using both models. Adaptation of the proposed testing method would enable researchers in the field to repeat experiments found in publications using these methods. By improving the throughput in sample production, more compositions or other parameters are accessible for rigorous study.

While other studies have focused on achieving low standard deviations in order to improve statistical power, here the even more basic problems associated with animal bone samples and their preparation are eliminated without being limited to testing on other clinically relevant materials, such as titanium or PEEK.

The most serious limitation of the test system is its intrinsic mechanical stability, limiting the adhesives that can be tested reliably to a maximum of roughly 2–3 MPa. By crosslinking the organic phase of the test substrate, the mechanical performance could be improved and consistency in surface gelatine concentration by eliminating its dissolution was achieved. Further and more detailed research regarding the functionalization and crosslinking reaction of the organic phase leaves room for additional improvements with respect to the mechanical performance of the system.

If desired, adhesive strength tests of experimental bone adhesives using the proposed methods can still be extended by repeating experiments on a natural bone-based test system as a complimentary method. This way, the merits of both approaches are used to their full potential.

## Supplemental Material

Supplemental Material - Biomimetic synthetic test system based on hydroxyapatite cement for adhesive strength evaluation of experimental mineral-organic bone adhesive materials Supplemental Material for Biomimetic synthetic test system based on hydroxyapatite cement for adhesive strength evaluation of experimental mineral-organic bone adhesive materials by Paul F Otto, Sebastian Hienz, Silvia Mittmann, Niklas Dümmler, Tobias Renner, Csaba Gergely, Juliane C Kade, Uwe Gbureck in Journal of Biomaterials Applications
