# Supplementary material for: Biomimetic synthetic test system based on hydroxyapatite cement for adhesive strength evaluation of experimental mineral-organic bone adhesive materials
Source: J Biomater Appl. 2024 Sep 9;39(6):566–77. doi: 10.1177/08853282241283537 (PMC11707973; doi:10.1177/08853282241283537)
Supplement: Supplemental Material - Biomimetic synthetic test system based on hydroxyapatite cement for adhesive strength evaluation of experimental mineral-organic bone adhesive materials [file sj-pdf-1-jba-10.1177_08853282241283537.pdf]

Supporting information

Table S1: Compositions and properties of mineral and mineral-organic test specimens as shown in Figure 1.

PLR [g/mL]	Gelatine solution concentration [wt%]	Compressive strength [MPa]	Resulting gelatine content [wt%]
2	10	3.13 ± 0.62	5.6
	30	0.56 ± 0.04	21.4
	40	0.58 ± 0.04	33.3
3	0	22.79 ± 4.33	0
	10	9.86 ± 2.00	3.7
	30	4.67 ± 0.35	14.3
	40	1.74 ± 0.57	22.2
4	30	10.98 ± 0.71	10.7
	40	5.99 ± 0.07	16.7
5	30	11.58 ± 1.16	8.6
	40	12.58 ± 2.26	13.3

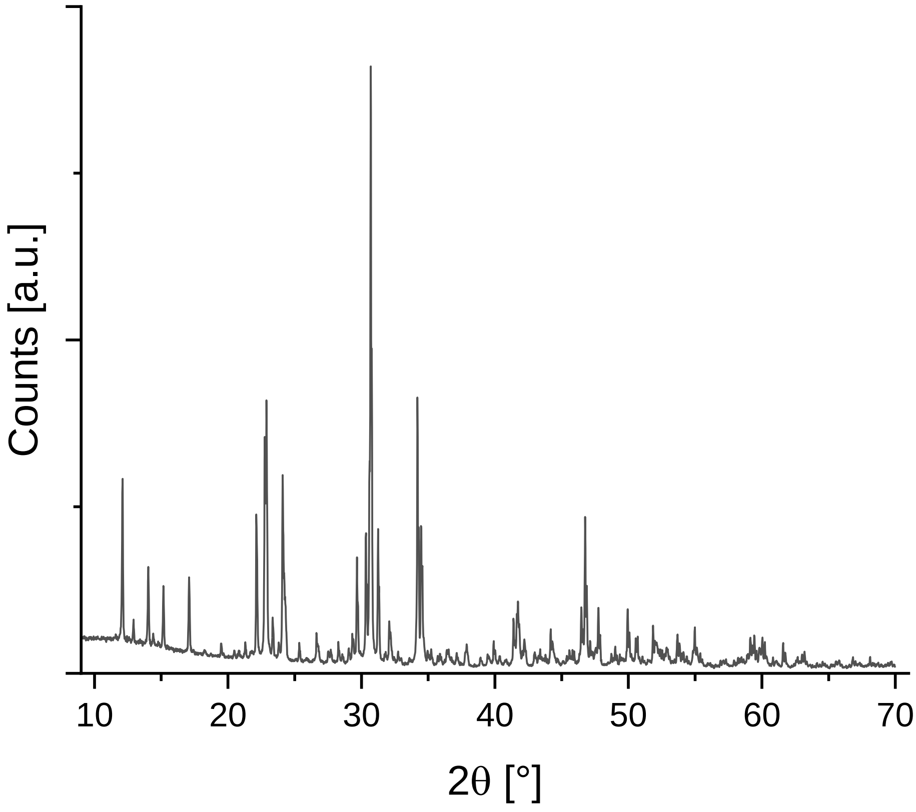

Figure S1: X-Ray diffractogram of α-TCP powder used for the preparation of mineral and mineral-organic test specimens. It has a phase purity of >99% determined by Rietveld refinement.

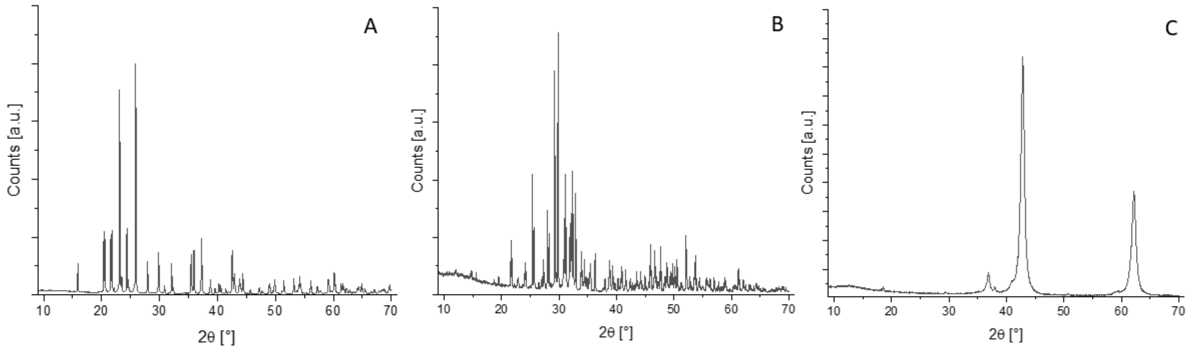

Figure S2: X-Ray diffractograms of powders **(A)** Farringtonite >99% **(B)** TTCP 94%, α-TCP 6% **(C)** MgO2933 99%.

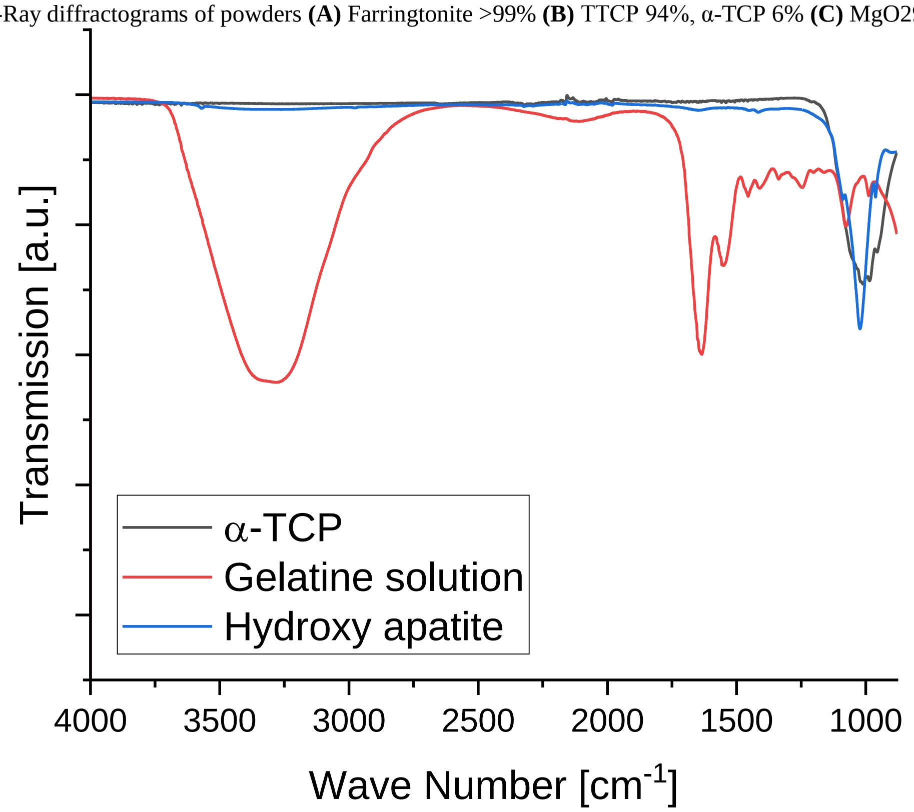

Figure S3. Reference FTIR spectra of pure α-TCP powder, gelatine solution and hydroxyapatite powder.

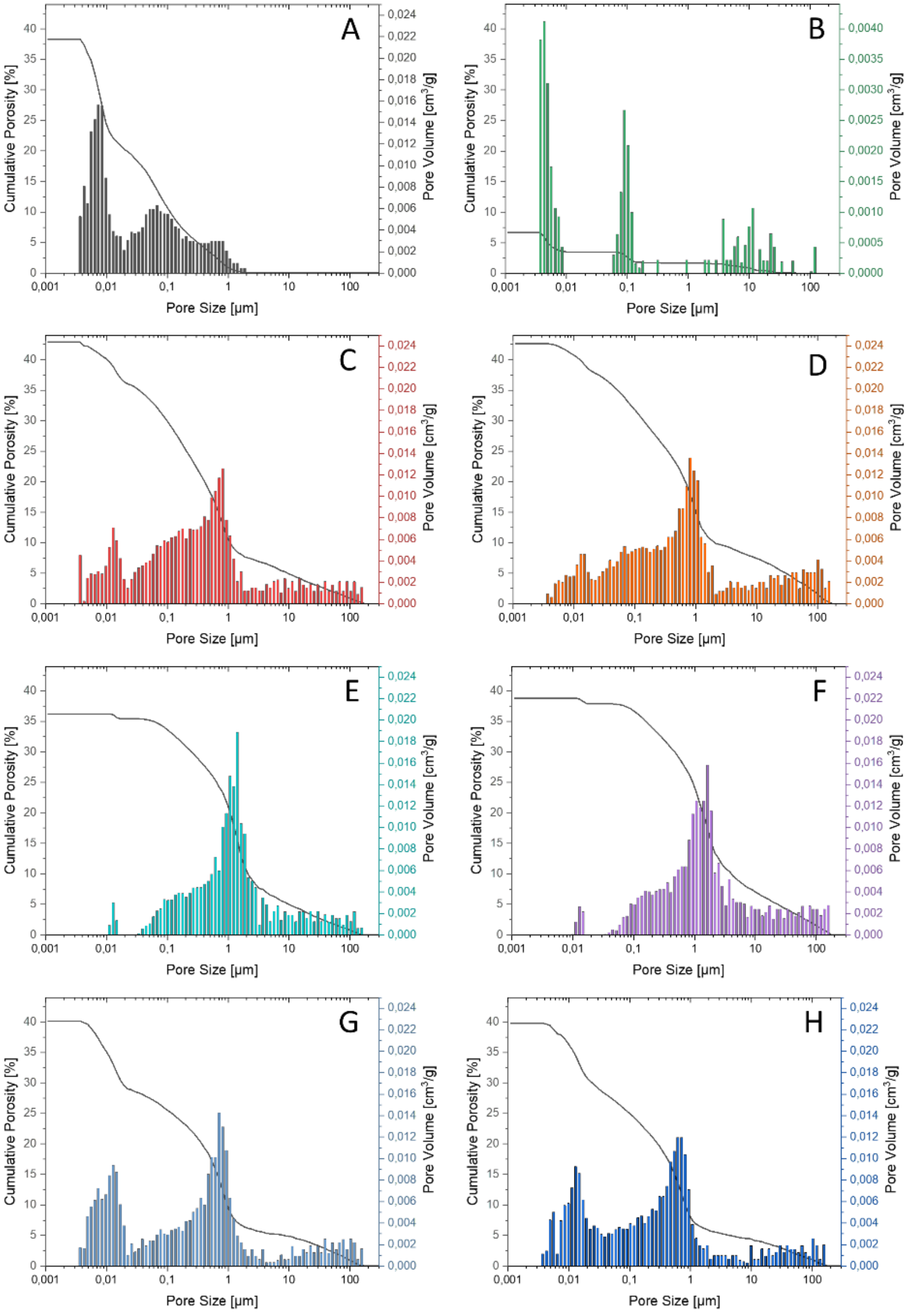

Figure S4: Porosity of test specimens prepared under various conditions. **(A)** purely mineral, **(B)** natural bone, **(C)** 3 days at 37°C, **(D)** 7days at 37°C, **(E)** 7 days at RT in 100% rel. humidity instead of water, **(F)** 14 days at RT in 100% rel. humidity instead of water, **(G)** 7 days at RT, (H) 14 days at RT.

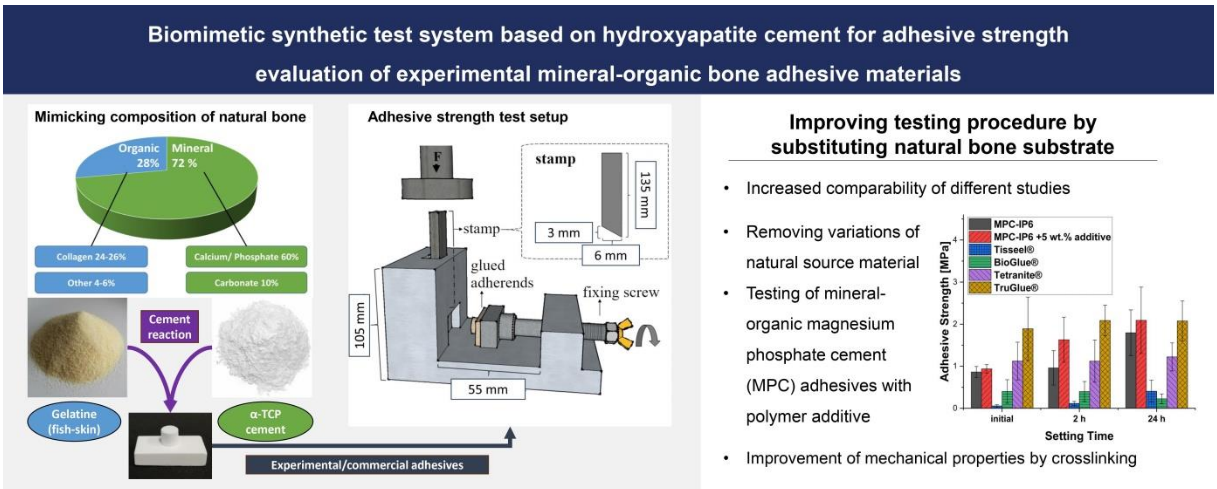
Biomimetic synthetic test system based on hydroxyapatite cement for adhesive strength evaluation of experimental mineral-organic bone adhesive materials
Mimicking composition of natural bone
Organic 28%
Mineral 72 %
Collagen 24-26%
Other 4-6%
Calcium/ Phosphate 60%
Carbonate 10%
Cement reaction
Gelatine (fish-skin)
α-TCP cement
Experimental/commercial adhesives
Adhesive strength test setup
F
stamp
stamp
3 mm
6 mm
135 mm
glued adherends
fixing screw
105 mm
55 mm
Improving testing procedure by substituting natural bone substrate
• Increased comparability of different studies
• Removing variations of natural source material
• Testing of mineral-organic magnesium phosphate cement (MPC) adhesives with polymer additive
• Improvement of mechanical properties by crosslinking
MPC-IP6
MPC-IP6 +5 wt.% additive
Tisseel®
BioGlue®
Tetranite®
TruGlue®
Adhesive Strength [MPa]
initial
2 h
24 h
Setting Time
